# Beta oscillations, timing, and stuttering

**DOI:** 10.3389/fnhum.2014.01036

**Published:** 2015-01-05

**Authors:** Andrew C. Etchell, Blake W. Johnson, Paul F. Sowman

**Affiliations:** ^1^Department of Cognitive Science, ARC Centre of Excellence in Cognition and Its Disorders, Macquarie UniversitySydney, NSW, Australia; ^2^Department of Cognitive Science, Perception in Action Research Centre, Macquarie UniversitySydney, NSW, Australia; ^3^Department of Cognitive Science, Macquarie UniversitySydney, NSW, Australia

**Keywords:** beta oscillations, stuttering, internal timing, basal ganglia, EEG/MEG

## Introduction

It has been proposed that one of the causes of stuttering is a deficit in brain timing networks (Alm, 2004; Ludlow and Loucks, 2004; Etchell et al., 2014). In stuttering, there appear to be structural and functional abnormalities in brain areas (such as the basal ganglia and supplementary motor area) that provide the substrate for internal timing (the ability to time movements without an external cue; Alm, 2010; Etchell et al., 2014). There are also structural and functional abnormalities in areas (such the cerebellum and premotor cortex) linked to external timing (the ability to time movements with an external cue), which are thought to represent compensatory plastic changes in stuttering (De Nil et al., 2008; Watkins et al., 2008; Lu et al., 2012). Currently, it remains unknown whether such deficits in internal timing mechanisms in stuttering may be manifest in any measurable neural marker. One possible candidate is oscillatory activity in the beta frequency band.

### The beta band and internal timing

Neural oscillations in the beta frequency band (15–30 Hz) are classically related to motor activity (see Kilavik et al., 2013 for review): decreasing in power prior to movement and then rebounding once the movement has finished (Pfurtscheller, 1981). Recently there has been considerable interest in the role beta oscillations might play in the brain's ability to represent temporal information because the observed associations between beta band power modulations and the timing of auditory beats (Arnal, 2012; Arnal et al., 2014). These investigations are only in their infancy but have already produced some intriguing observations. For example, Fujioka et al. (2012) used magnetoencephalography (MEG) to measure beta oscillations while subjects passively listened to sounds at regular (390, 585, and 780 ms) and irregular intervals (varying between 390 and 780 ms). Whereas the slope of the decrease in beta power after the onset of sounds was identical across conditions, the rising slope of beta power was maximal prior to the onset of the next expected sound for the regular but not the irregular conditions. The authors concluded that modulations in beta oscillatory activity represented an internalization of predictable intervals between sounds. More recently, Cirelli et al. (2014) replicated these results in an electroencephalography (EEG) study showing a similar pattern of anticipatory beta activity across multiple temporal intervals. Arnal (2012) contends that the beta modulation observed in the Fujioka et al. (2012) study may reflect the motor system generating efference copy signals at the tempo of stimulation. Empirical support for this prediction comes from recent work by Arnal et al. (2014) who showed that correctly judging whether or not a target tone had been delayed in time was associated with greater cortical beta power before the target tone.

There is good evidence to suggest that beta oscillations in the cortex reflect oscillatory activity originating in subcortical structures. Much of our knowledge of beta oscillatory activity in subcortical regions comes from studies in animals or humans with deep brain implants to treat Parkinson's disease (e.g., Levy et al., 2000) because it is not routinely possible to make such invasive recordings in healthy adults. Nevertheless, the pattern of beta desynchronization and resynchronization observed in the cortex during and subsequent to movement can also be observed in the basal ganglia of humans (Brittain and Brown, 2014) and macaques (Courtemanche et al., 2003). MEG experiments indicate the basal ganglia and cortical regions are connected via functional loops (see Jenkinson and Brown, 2011) further suggesting there is a relationship between beta oscillations at different levels of the brain. Consistent with this line of reasoning, Klostermann et al. (2007) reported that in humans, beta band power recorded from the basal ganglia (using depth electrodes) and the scalp (using EEG) during a cued choice reaction time task was correlated in phase and amplitude (measured by magnitude-squared coherence). Likewise, it has been demonstrated experimentally that the cortex and the subthalamic nucleus exhibit beta band amplitude and phase coherence, and it is hypothesized that such an interaction relies on the striatum (Hirschmann et al., 2011).

The relationship between cortical and subcortical beta oscillations, together with the fact that beta oscillations in the motor and auditory cortices are related to internal timing (Fujioka et al., 2012), suggests that beta oscillations in the striatum might also be related to internal timing. Accordingly, Bartolo et al. (2014) examined the role of beta oscillations in timing by recording local field potentials from microelectrodes implanted in the putamen of healthy macaques during a synchronization and continuation task. This task requires that the macaques tap in time with a beat (the synchronization phase) and that they continue to tap once the beat has been removed (the continuation phase). Whereas the synchronization phase is an index of external timing (due to the presence of an external stimulus), the continuation phase is an index of internal timing (due to the absence of an external stimulus; Teki, 2014). The main finding from the Bartolo et al. (2014) study was that beta activity was strongly biased to the continuation phase as opposed to the synchronization phase of the task indicating that putamenal beta oscillations are tuned to internal rather than external timing of movement.

There is evidence that beta oscillations can be recorded from the striatum during self-paced movements in humans. Intracranial recordings from the putamen of an epileptic patient showed that beta power peaks near the onset of self-paced bimanual finger extensions (Sochurkova and Rektor, 2003). While not focusing directly on beta oscillations, there is evidence from functional neuroimaging to implicate the striatum in internal timing in healthy adults. For example, Grahn and Rowe (2013) demonstrated that the putamen responds to the detection of regularity rather than the detection of beats, suggesting that it is involved in internally paced movement rather than simply the detection of the presence or absence of a beat. The basal ganglia are also more active during subjective judgments of temporal intervals relative to judgments of externally timed intervals (Coull et al., 2013) and the putamen shows greater activity during continuation tapping but not synchronization tapping as compared to rest (Rao et al., 1997). Interestingly, individuals with bilateral lesions to the basal ganglia perform poorly on the continuation but not the synchronization phase of a rhythmic tapping task (Coslett et al., 2010). Such evidence suggests that the putamen is essential for internal timing.

### The beta band and stuttering

What are the implications of these results in the context of stuttering? If indeed stuttering is a disorder of internal timing (Alm, 2004; Etchell et al., 2014), and if beta oscillations in the basal ganglia are involved in internal timing (Bartolo et al., 2014) and/or the cortex (Fujioka et al., 2012; Cirelli et al., 2014) then it follows that stuttering could be a disorder caused by striatal abnormalities that result in abnormal beta power. More specifically, stuttering could be a disorder in which beta power is hypoactive or where the relationship between cortical and subcortical beta power is unstable. That there are exaggerated beta band responses in adults who stutter (AWS; Rastatter et al., 1998) and reduced beta band responses in children who stutter (CWS; Özge et al., 2004) provides some evidence for this contention.

The suggestion that stuttering is a disorder caused by abnormalities of the striatum is consistent with neuroimaging studies of CWS. Investigating differences in brain structure and function of CWS is valuable because they have had much less time to react to stuttering as compared to AWS. Due to the young age of the population, any differences observed between CWS and children who do not stutter (CWDS), are more likely to reflect anomalies related to the cause of stuttering rather than consequences of stuttering (see for review Chang and Zhu, 2013; Etchell et al., 2014; Sowman et al., 2014). The striatum is involved in the articulatory control of speech at different rates (Wildgruber et al., 2001; Riecker et al., 2005, 2006) and in speech rhythm (Fujii and Wan, 2014) and research shows CWS exhibit reduced levels of connectivity between the putamen and several cortical structures including the supplementary motor area, superior temporal gyrus and inferior frontal gyrus (Chang and Zhu, 2013). CWS also have less gray matter in the left putamen (Beal et al., 2013) than CWDS. Interestingly one study reported CWS exhibit reduced levels of beta band activity at rest in the cortex compared to CWDS (Özge et al., 2004).

If abnormal beta power arising from the striatum is causally related to stuttering, then fluency inducing manipulations should normalize beta power. This contention is supported by functional neuroimaging and electrophysiological studies. The finding that putamenal beta band oscillations are biased toward internal timing (Bartolo et al., 2014), together with the fact that the putamen responds to regularity (Grahn and Rowe, 2013) and is known to exhibit beta band oscillations (Sochurkova and Rektor, 2003), suggest that the striatum tracks regular sounds via modulation of beta activity. An fMRI study has shown that AWS exhibit less activation of the basal ganglia during normal speech compared to rest, but that when speaking in time with regular sounds, the level of basal ganglia activation is comparable to adults who do not stutter (AWDS; Toyomura et al., 2011). Given the positive relationship between BOLD activity and beta band responses (Laufs et al., 2003), the normalization of striatal activity may perhaps be accompanied by normalization of beta band activity. Additionally, since regular sounds influence cortical beta power (Fujioka et al., 2012; Cirelli et al., 2014) and cortical beta is associated with subcortical beta oscillations (Klostermann et al., 2007; Jenkinson and Brown, 2011), it is likely that regular sounds also influence beta power in subcortical structures. There is evidence that delayed auditory feedback (DAF), another fluency inducing mechanism, alleviates cortical beta band abnormalities in AWS. Rastatter et al. (1998) used EEG to show that AWS exhibit hyperactivity of the beta band in the cortex when reading aloud. This hyperactivity was markedly reduced by DAF. In the same way that a metronome affected the haemodynamic response in cortical and subcortical structures (Toyomura et al., 2011), DAF might have also affected beta band oscillations in both cortical and subcortical structures. Indeed most fluency inducing mechanisms seem to work by facilitating coupling between auditory and motor systems as well as the putamen (Stager et al., 2004).

It is unclear whether the hyperactivity of the beta band activity in stuttering (Rastatter et al., 1998) reflects causal or compensatory mechanisms. Since the volume of white matter and beta band amplitude increases with age (Uhlhaas et al., 2010) and because the density of the white matter fibers underlying the motor cortex and superior temporal areas were negatively correlated with the severity of stuttering (Cai et al., 2014). It is our opinion that the hyperactive beta oscillations in the cortex reported in Rastatter et al. (1998) may be compensating for *hypoactive* beta oscillations in the basal ganglia. DAF may have normalized the beta band oscillations in the basal ganglia thereby reducing the need for compensation via hyperactive beta in the cortex. This idea suggests both AWS and CWS should exhibit reduced beta band responses in the putamen when internalizing rhythms. The fact that fluency-inducing mechanisms reduce the hyperactivity of the beta band in the cortex has major implications for stuttering. Firstly, it implies that without regular external stimulation, AWS have abnormal beta oscillations in the cortex and possibly the striatum. Secondly, normalizing compensatory hyperactivity in the cortex as well as temporarily alleviating stuttering implies that DAF may act to normalize hypoactive oscillations in the striatum.

In summary, if stuttering is a disorder of internal timing and internal timing is represented by modulations of oscillatory power within the beta band in the striatum, then it is likely that the cause of stuttering is reflected in abnormal beta band oscillations in the putamen. This is consistent with the structural and functional abnormalities in CWS (Beal et al., 2013; Chang and Zhu, 2013), the notion that beta band oscillations are evident in the putamen (Sochurkova and Rektor, 2003) and that CWS exhibit beta band abnormalities (Özge et al., 2004). The idea that beta oscillations reflect the neural abnormality causing stuttering is further supported by the observation that fluency-inducing mechanisms normalize activity in the putamen (Toyomura et al., 2011) and also beta power in the cortex (Rastatter et al., 1998). Future studies should thoroughly investigate beta oscillations in stuttering.

### Conflict of interest statement

The authors declare that the research was conducted in the absence of any commercial or financial relationships that could be construed as a potential conflict of interest.
